# COVID-19’s gendered effect on subjective wellbeing in MENA countries

**DOI:** 10.1038/s41598-024-84452-7

**Published:** 2025-01-10

**Authors:** Ghada Barsoum, Mahdi Majbouri

**Affiliations:** 1 The American University in Cairo, Cairo, Egypt; 2 Babson College, Wellesley, USA

**Keywords:** Psychology, Risk factors

## Abstract

The COVID-19 pandemic has been a time of great intensity that exposed many existing inequities in facing this global threat. Building on Galtung’s conceptualization of positive peace as the absence of structural violence and institutionalized inequality, we study the gendered effect of the COVID-19 pandemic on reported subjective wellbeing (SWB) in four countries in the Middle East. Data from mobile phone panel surveys, with a total sample of 12,614 observations collected during this critical juncture, show that women consistently reported a lower level of SWB than men in all four countries. Women experienced higher increases in unemployment rates than men in all four countries, despite their already higher rates prior to the pandemic. Controlling for individual characteristics and geographic-time fixed effects, the main factor associated with lower SWB was the decline in household income, reflecting the intersectionality of poverty and gender. In addition, the reported increase in the time spent on housework negatively affected women’s SWB, particularly in urban areas. The pandemic has further accentuated gender inequality in all four countries and exposed the inherent structural violence experienced by women in these contexts.

## Introduction

The streets of Cairo, normally a bustling sleepless city, were silent. Curfew time extended from 7:00 PM to 6:00 AM in March 2020, with shifting hours throughout the summer and fall of 2020. Cairenes, like millions of others around the world, were locked at home. With a population density that exceeds 20,000 people per square kilometer in some areas^1^, it is not hard to imagine how densely crowded some of these homes were. Even in the hours before the curfew, children were not going to school,some working family members were returning home earlier than usual; others were working from home or lost their work altogether. The longer these members stayed at home, the longer the hours needed for care work, with the brunt of the housework falling squarely on the women in the house. By all means, the pandemic lockdown was a time of great intensity^2^. It also had serious repercussions on individuals’ subjective wellbeing (SWB), which is commonly used in research as a direct indicator of psychological wellbeing (e.g.,^3^). The pandemic impacted some of the long-held factors affecting SWB: income, healthy life expectancy, social support, prevalence of generosity and freedom of choice^4^. The burden of the disease and lost lives (at least 1.8 million, potentially 3 million as excess mortality in 2020 alone according to World Health Organization^5^ has been compounded by the intensity of the lockdown, the economic repercussions of the pandemic, and the increasing burden of care work on women. This paper seeks to capture this dynamic with a focus on four countries in the Middle East: Egypt, Jordan, Morocco, and Tunisia. The analysis situates the data on this dynamic in the literature on structural violence and positive peace^6–8^.

The modern history of the Middle East is rife with ongoing and rivaling conflicts, which have consumed researchers’ energy in trying to understand their complexity and historical underpinnings (e.g.,^9,10^). Little to no research has sought to look at positive peace, or the institutions and structures that sustain peaceful societies^6^. The conceptualization of positive peace does not place war and peace as binary outcomes, but rather as two opposites on a continuum^7^. Equality values, particularly gender equality, are central to achieving quality and positive peace^8^. The Middle East region has one of the lowest female labor force participation rates in the world^11^, and one of the largest gender gaps in vulnerable employment^12^. Survey data consistently shows the prevalence of inequitable gender attitudes^13^. Women’s oppression cuts through the fabric of society, breading intolerant values in other aspects and reinforcing lower-quality peace^8^. SWB, similar to other contexts, is ultimately grounded in these institutional arrangements^3^.

The COVID-19 pandemic has been a critical juncture that exposed many existing inequities in facing this global threat. The obvious healthcare system inequities and governance capabilities for mass vaccination aside, the pandemic has particularly exposed the poor research infrastructure in the global South. Much of the research that sought to capture the effect of this historical moment came from the Northern hemisphere. Aside from reports by some international organizations, a few academic studies emerged about the global South (e.g. Seck et al.^14^ on Asia–Pacific countries,İlkkaracan and Memiş^15^ on Turkey, Mansour and Benmouro (2023) on Morocco; and Desai et al.^16^ on India). This paper seeks to contribute to this sparse literature by focusing on the Middle East and North Africa (MENA) region, a particularly understudied region from many social science aspects including positive peace and SWB. By no means do we seek to claim that the results of this study are representative of the whole region. The diversity of the economic situation of the countries in the Middle East, like everywhere else, has translated into a diversity of responses to the health and economic repercussions of COVID-19. However, Arab countries in MENA remain oddly unified in women’s lower-than-global-average rates of labor force participation and political representation and in the prevalence of conservative gender norms^11^.

Utilizing a unique dataset from mobile phone panel surveys with a total sample of 12,614 observations, we study the gendered impact of COVID-19 on SWB. In other words, how the pandemic affected the two genders differently. We show that the pandemic has further exposed the gender inequities in these countries, farther moving them away from positive peace. The pandemic provided additional stressors on men and women that constituted a grave form of structural violence, negatively impacting SWB and the quality of peace in these contexts. Our analysis shows that women consistently reported a lower level of SWB than men in all four countries. While education was positively associated with an increase in SWB, this was only for men. Women experienced not only worse labor market outcomes, but also more burden of work at home (40% of women reported a rise in the time spent on childcare and housework). Women’s unemployment-to-population ratios rose during the pandemic and reached two to three times their levels before the pandemic. This may show that as households experienced declines in their income during the pandemic, women sought employment to raise household income to pre-pandemic levels adding an additional stressor to this group.

A decline in household income was a significant determinant of women’s and men’s lower SWB, which has been documented elsewhere (e.g.^4^). Controlling for administrative zone-time fixed effects (a robust specification that removes any confounder that is specific to an administrative zone within a country at the time of a survey) and individual demographic characteristics (age, education, household size, etc.), men and women whose households experienced an income decline reported about 18% and 10% reduction in their SWB, respectively. This decline was particularly pronounced for urban women compared to rural women, presumably because employment patterns were affected more in urban areas due to stricter lockdown restrictions. A larger share of poorer households experienced a decline in their income than those in higher income quintiles due to the pandemic (compared to their income in February 2020), which shows the greater impact of the pandemic on the poor and the intersectionality of gender and poverty. However, a significant determinant of women’s SWB has been the rise in the hours spent on childcare and housework during the pandemic. This particularly affected urban women. Those urban women who reported doing more housework during the pandemic than before it, experienced a 8.7% decline in their SWB. We did not find any association between other controls (age, marital status, living in an urban area, and household size) and SWB for both men and women.

Following this introduction, section two provides a background on the policy response to the pandemic in the four countries under study. The conceptual framework for analyzing the results follows. Section four presents the data and the methodology, including a discussion on mobile phone surveys as a data collection tool and its impact on sampling bias. Section five presents the results of survey data analysis on women’s conditions during the pandemic, and we conclude by further situating this data within the analytical framework of structural violence and positive peace.

## Background: the policy response to COVID-19 and the region’s gendered realities

The policy response to COVID-19 in the four countries under study followed a similar pattern. By the third week of March 2020, all four countries imposed partial lockdowns following varying hours. In Egypt, for example, the curfew hours lasted from 7 PM to 6 AM and were gradually eased to start by midnight in July 2020^17^. Despite the relatively relaxed lockdown measures, compared to countries in the global North, the impact on the labor market has been quite strong. Krafft et al.^17^ show that many wage workers in all four countries under study, specifically those in the informal economy, have lost their jobs or had to work reduced hours at lower earnings due to COVID-19. The majority of employers and the self-employed also reported that their revenues in 2020 were less than 2019. Moreover, almost half of households in the four counties reported a decrease in their income due to the pandemic. This was particularly experienced by poorer households (ibid.).

National statistics in all four countries confirm these patterns. In Egypt, the country’s central statistical bureau announced that the unemployment rate increased to 9.6% in the second quarter of 2020, up from 7.7% in the first quarter^18^. The labor force contracted from 29 million in the first quarter, to 26.6 million in the second quarter, an 8% decrease (ibid.). The outcome was also highly gendered: while male unemployment rose to 8.5% (up from 4.5% in the first quarter), female unemployment fell to 16.2%, down from 21.9% in the first quarter. It is probably the case that some unemployed women became discouraged from searching for jobs, given the complexity of the lockdown and job scarcity. Similarly, the Department of Statistics in Jordan announced that the unemployment rate reached (24.7%) during the fourth quarter of 2020,representing an increase by 5.7 percentage points from the fourth quarter of 2019^19^. Unlike Egypt, women’s unemployment rates showed worsening results in Jordan compared to men’s. The unemployment rate for males reached 22.6% during the fourth quarter of 2020 against 32.8% for females. It became clear that the unemployment rate increased for males by 4.9 percentage points and for females by 8.7 percentage points compared with the fourth quarter of 2019^19^. In Tunisia, unemployment increased from 15% prior to the pandemic to 17.8% by the end of the first quarter of 2021. Moreover, it continues to affect women (24.9%) and young people aged 15–24 (40.8%) in particular^20^. In Morocco, the unemployment rate rose from 8.1 to 12.3% between 2019 to 2020. The male unemployment rate rose from 7.2 to 11.3%, and the female unemployment rate increased from 11.1 to 15.6%^21^.

The social policy response in all four countries included interventions that by far did not match the intensity of the impact of the pandemic. In all four countries, there have been cash transfers to informal workers and the expansion of the outreach of programs of social assistance programs. In Egypt, the government increased pensions by 14%; expanded the outreach of its cash transfer program (*Takaful* and *Karama*), and provided cash transfers to 1.6 million irregular workers. Jordan reduced social insurance contributions and offered temporary cash transfers to daily workers. Morocco offered cash transfers to those who lost their jobs and extended access to health care insurance to informal workers; and Tunisia offered additional pension payments to households. Similar to most, if not all countries of the world, none of the four countries offered women-specific interventions or addressed the challenges of the increase in care responsibilities on women.

However, the situation of women in the region was already highly vulnerable. Despite positive trends in education and health, women’s employment status and labor force participation in the region remain the lowest globally (World Bank, 2024). Patriarchical views about gender roles and women’s inequitable rights have long been documented in the region (e.g., UNDP 2002;^22,23^). Despite discursive policy support and advances in the realms of education and health gendered outcomes^24^, there continues to be a documented resistance to women’s work outside the house and their participation in aspects of political and public life (e.g.^25^). A staggering 98% of men and 88% of women responding to a survey conducted in three countries in the region agreed to the statement that when work opportunities are scarce, men should have access to jobs before women (El Feki^13^: 50). Corresponding with these results, women’s share of household chores is quite inequitable as the same study shows, with only 26% of ever-married men reporting ever carrying out tasks related to washing clothes or cleaning the house. Selwaness and Helmy^26^ show that married women spend seven times as much time on unpaid care work as married men in Egypt. Moghadam^27^ notes that the pandemic has also been widely reported as having an especially negative impacton women’s employment.

## Structural violence, positive peace and subjective wellbeing: a framework for analysis

Violence, argues Galtung^6^ (168), is “present when human beings are being influenced so that their actual somatic and mental realizations are below their potential realization”. This definition squarely places psychological wellbeing to positive peace, understood as an absence of structural violence (ibid.). It is “structural” because it is embedded in the political and economic organization of society, and it is “violent” because it is likely to cause hurt to the people affected by it (ibid.). Although primarily focused on physical violence, Gilligan^28^ argues that structural violence is normalized in social institutions and is manifest in differential access to resources. Farmer et al.^29^ place structural violence in social injustices, bringing extreme and relative poverty, racism, and gender inequality to the fore as forms of violence. Because peace and conflict are best conceptualized as two ends of a continuum, developing or transitional countries are typically farther from a peace position on this continuum due to grave inequities^7^.

The study of gendered access to health and wellbeing has increasingly utilized notions of structural violence and positive peace as a prism for the analysis of gender inequality (e.g.,^30^). The concept has allowed for the consideration of the extent to which people’s lives and health outcomes are affected by institutionalized inequalities and intersectionalities of gender, poverty, racism and other manifestations of inequality. Studies on vulnerable women groups in the global South (e.g.,^31–33^), and the global North (e.g.,^34^) sought to explain women’s wellbeing deficit from the lens of structural violence.^29^ article connecting structural violence to clinical medicine is attributed for serving as an antecedent to a large ensuing body of research taking this approach^30^. Melander^8^ builds on Galtung and others to depart from mainstream analyses of gender and conflict with their view of women solely as victims (see^35^ for a critique of this). He places particular value on the role of gender equality in achieving quality peace. Melander^8^ argues that when equality values are compromised and women’s oppression is an accepted norm, this breeds intolerance and the normalization of injustices and reinforces lower-quality peace.

The analysis in this paper shows that the COVID-19 pandemic has been a critical juncture that exacerbated gender and income inequalities and further exposed the structural violence women experience both in the public sphere and in the intimate setting of the family, affecting their SWB. Literature has shown that the pandemic impacted some of the long-held factors affecting SWB (e.g.^4^). Focusing on Germany, Zacker and Rudolph^36^ whistled one of the early alarms that the COVID-19 pandemic represents not only a medical and economic crisis, but also a psychological crisis related to the decline in people’s SWB. Using longitudinal data, Helter et al.^37^ show that the levels of wellbeing and mental health decreased for most respondents across the three lockdowns in Austria.

Research on SWB prior to the pandemic supported these findings. For example, Mata et al.^38^ highlighted the negative impact of reduced physical activity, while Blom et al.^39^ highlighted the negative impact of employment challenges on straining the relationship between couples and their wellbeing. Earlier research has also documented the negative effect of prolonged screen time, a key feature of the lockdown, on the health and wellbeing of children and adolescents^40^. The specific effect of the economic crisis on SWB received particular research interest. For example, Möhring et al.^41^ show that there has been a general decline in family and work satisfaction in Germany. This research also highlights the accumulated knowledge of the effect of economic hardships on life satisfaction (e.g.^42^) and the impact of job quality indicators on SWB^43^.

Research on the role of gender in SWB, which almost exclusively comes from the global North, has long shown that women and men have similar levels of SWB (e.g.^44^). In fact, studies by Tay et al.^45^ and by Blanchflower and Oswald^46^ argue that women tended to have higher levels of life satisfaction than men. Inglehart^47^ qualifies this gender-happiness dynamic by addressing the role of age, noting that women under 45 tended to be happier than men, but older women were less happy based on a study with a sample of 146,000 respondents from 65 societies. But COVID-19 seems to have broken this long-held assumption, by negatively affecting women’s wellbeing in some unprecedented ways. For example, Möhring et al.^41^ show that the decrease in family and work satisfaction during the pandemic was more pronounced for mothers than fathers, reflecting the burden of care work on women. Similarly, Collins et al.^48^ looked at the gender gap in working hours during the pandemic and showed how school and daycare closures increased caregiving responsibilities for mothers, particularly those with young children, and reduced mothers’ work hours four to five times more than fathers in the context of the United States. Craig and Churchill^49^ documented the increased burden of unpaid work for women during the lockdown,and that while men contributed more to care work, their share was at the level women were doing before the pandemic. More seriously, the incidence of domestic violence increased with the lockdown (e.g., Hsu and Henke^50^), a less documented situation in the global South due to lack of data, including in this study.

From the sparse literature on the global South, İlkkaracan and Memiş^15^ document the doubling of women’s already long time on care work in Turkey. They also note that employed women saw what the researchers describe as “an alarming intensification” in their workload that would make it hard for these women to sustain a decent work-life balance. Desai et al.^16^ looked at the impact of COVID-19 on women wage workers, noting that women experienced greater job losses and highlighting the gendered impact of this macro crisis in India. Seck et al.^14^ use evidence from eleven countries in Asia–Pacific to show that women were disproportionately shouldering the burden of unpaid care and domestic work triggered by the lockdowns, at the expense of a faster rate of losing livelihoods than men. They also document a disproportionate worsening of women’s mental health. Earlier research supports the interplay between gender ideologies, amount of time availability, and resource dependence on the perception of fairness in the division of work among women^51,52^.

While working from home has been an option for many in the global North during the pandamic lockdown, this was the case for only a fraction of workers in the global South^53^. The experience of essential workers in the global North (e.g.,^54^) simply resonated with much more people in the global South. In low-income countries, only one of every 26 jobs could be done from home according to Garrote Sanchez et al.^53^. This further aggravated the structural violence associated with the pandemic in the global South. Unable to work from home, more workers were forced to choose between the health threats of a pandemic and their livelihood opportunities. This could have had a substantial impact on people’s SWB in the global South that remains under-reported by the burgeoning research on the effect of COVID-19.

## Data and methodology: a gendered critique of mobile phone sampling

The COVID-19 MENA Monitor Surveys (OAMDI^55^, publicly available at www.erfdataportal.com), the source of data for this study, were collected by phone during the pandemic in four countries: Egypt (two waves), Jordan (one wave), Morocco (two waves), and Tunisia (two waves). The first wave of the survey in Egypt was conducted in June 2020 (about four months into the pandemic). Later in the year, the first waves of the survey in Morocco and Tunisia were collected in October 2020. In February 2021, the second waves of the survey in Egypt, Morocco, and Tunisia, as well as the first wave of the survey in Jordan were conducted. The surveys covered demographic characteristics, labor market outcomes, such as employment status, economic activity and income, SWB, and women’s work at home during the pandemic. All surveys, except the Egyptian first wave, contained questions on SWB. Therefore, we use all survey data, except the Egyptian first round, in this study.

The sample universe for the household surveys was mobile phone users aged 18–64. Random digit dialing (RDD) within the range of valid numbers was used, with up to three attempts. Samples were stratified by country-specific market shares of mobile operators. The number of observations in each county-wave is at least 2000, except for the first wave of the Egyptian survey, which we do not use (There are 1923 observations in the Egyptian first wave, but 2000 observations in its second wave. Jordanian first wave has 2549 observations. The sample sizes in the Moroccan first and second waves are 2007 and 2002 and in the Tunisian first and second waves are 2000 and 2077). Survey data was collected from one individual in a household. The surveys collected information about the respondent only but not the other members of the household. Observations are weighted, and the weights were calculated based on the following dimensions: (1) Telephone operators and their market shares, provided by the data collection firm, (2) The number of phones by each operator for individuals (individual weight) and household members (household weight and household member weight), and (3) Representative data with comparable demographic and household characteristics (to weight for non-response).

It is important to be cognizant of the gender differences in phone ownership and autonomy of use. The “mobile gender gap” as came to be dubbed in the literature is a key factor^56^. Women in low- and middle-income countries are 20 per cent less likely to use mobile internet than men (ibid.). This explains why the women interviewed using RRD tended to be of a higher socio-economic status than the men, due to the skewed distribution of mobile phones. While sample weighting takes into account observable variables of demographic and household characteristics, weighting cannot take into account levels of unobservable variation in empowerment and autonomy. The critical feminist tradition has long learned from Virginia Woolf’s notion of a woman’s “room of her own”^57^) The mobile is a modern representation of women’s ability to have the time, the resources, and the autonomy to freely talk to a stranger who is asking about the impact of COVID-19. Consistently, the sample for the survey in the four countries had fewer women than men. This is only less pronounced in Jordan, but the difference remains drastic in other countries.

To address this sampling bias, our decision has been to consider the men and women as two separate samples with different characteristics.Although results were weighted on observable characteristics to ensure comparability to mobile phone users in in-person surveys, results only generalize to the universe of mobile phone users, who are disproportionately higher income, male, and more educated. Table A6 reports the differences between men and women; one interesting fact to note is that about 50% of surveyed women were in the labor force (either employed or unemployed), which is larger than the national statistics for women. As we discuss below, we try to capture as many unobservable characteristics (such as gender norms, gender equality, societal attitudes, culture, socio-economic conditions in a geographic region at a specific time, and government policies to name a few) as possible with geographic-time fixed effects. However, one should note that fixed effects do not necessarily solve sample selection on unobservable characteristics. They would solve some endogeneity problems.

We pick the most relevant variables in these datasets to study the impact of the pandemic on women and address the research question on the gendered effect of COVID-19 on SWB and its determinants (See Appendix Table 6 for summary statistics for women and men in the sample). Almost all variables we use were readily available in the dataset. SWB, however, was embedded in five questions (As mentioned before, these questions were not asked in the Egyptian first wave of the survey (that is, June 2020 Egyptian survey), but they were asked in the Egyptian second wave in Feb. 2021, Jordanian first wave in Feb. 2021, and both waves of Moroccan and Tunisian surveys (Nov. 2020 and Feb. 2021). They specifically ask how often (never to all the time) a respondent experienced the following: (1) felt cheerful and in good spirits, (2) felt calm and relaxed, (3) felt active and vigorous, (4) woke up feeling fresh and rested, (5) her/his daily life has been filled with things that interest him/her. The responses to these questions by sex are reported in Tables A1–A5 in the Online Appendix. Unfortunately, the survey is missing some key issues related to gender-based violence, which has been repeatedly highlighted in other studies and can have a detrimental effect on women’s SWB.

**Fig. 1:**
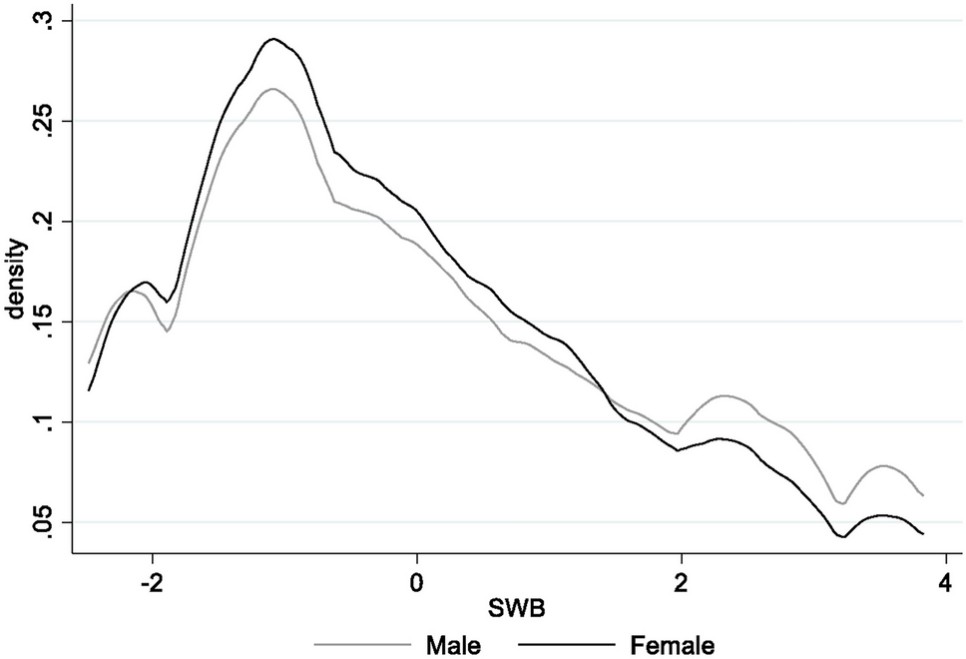
Distribution of Subjective Wellbeing Index by Sex. Authors’ calculations. The sample sizes for women and men before the pandemic are 7528 and 5107, respectively.

Using the principal component analysis, we created the SWB composite index (SWB) based on the responses to the above five questions. The lower the SWB index for a respondent, the less often she/he experienced the five statements above; in other words, the unhappier the respondent was. As the summary statistics in Table 1 show, the SWB index is a number between − 2.48 and 3.82; its average is zero, and its median is − 0.35. Figure 1 depicts the distribution of the SWB index for women and men. It shows that women, in general, had lower SWB than men. The average SWB index for women is about 0.1 standard deviation (0.185 units or 7%) smaller than the average SWB index for men. Unfortunately, it was not possible for the designers of the surveys to collect data on these questions before the pandemic. So SWB of respondents before the pandemic is unknown.

**Table 1:** Summary Statistics (N = 12,614).

Variables	Mean	Median	St. Dev	Min	Max
Subjective wellbeing index	0.00	− 0.35	1.71	− 2.48	3.83
Normalized subjective wellbeing index	0.00	− 0.21	1	− 1.45	2.24
Household income decreased	0.53	1	0.50	0	1
*Labor Force Status*					
Employed	0.51	1	0.50	0	1
Unemployed	0.22	0	0.42	0	1
Out of labor force	0.27	0	0.44	0	1
*Education*					
Less than basic	0.27	0	0.44	0	1
Basic	0.18	0	0.39	0	1
Secondary	0.32	0	0.47	0	1
Higher education	0.23	0	0.42	0	1
Age	37.00	35	12.16	18	64
Married	0.65	1	0.48	0	1
Urban	0.70	1	0.48	0	3
Household size	4.87	5	2.35	1	54
More childcare vs. Feb. 2020*	0.27	0	0.44	0	1
More housework vs. Feb. 2020*	0.33	0	0.47	0	1

Subjective Wellbeing Index is formed from principal component analysis of five variables described in Tables A1–A5. Household Income Decreased is a binary variable that is one if household’ income declined and zero otherwise. Employed, Unemployed, and Out of labor force are binary variables that are equal to one if a respondent was employed, unemployed, or out of labor force, respectively, and zero otherwise. Less than basic, Basic, Secondary, and Higher education are binary variables equal to one if a respondent’s education is at the associated level and zero otherwise. Married is a binary variable equal to one if a respondent was married at the time of the survey and zero otherwise. Urban is a binary variable equal to one if a respondent lives in an urban area.

*More childcare vs. Feb. 2020 and More housework vs. Feb. 2020 are binary variables equal to one if a woman spent more time on these activities in the week prior to the survey relative to Feb. 2020. Since these questions are only asked of women, the number of observations for those is 5100.

We start our analysis by studying the basic statistics of employment, unemployment, the burden of work at home for women, and SWB across income, time, and countries. This allows us to identify some interesting patterns in the data. In the final step, we run regressions, as described by the following equation, to find associations between SWB and other relevant variables in the dataset:

1 $${Y}_{ijt}=\sum_{z=1}^{K}{\beta }_{z}{X}_{ijt}^{z}+{\gamma }_{jt}+{\varepsilon }_{ijt}$$

In which ${Y}_{ijt}$ is the SWB index for individual $i$ in the administrative zone $j$ at time $t$; ${X}_{ijt}^{z}$ is a set of $K$ individual characteristics for individual $i$ in the administrative zone $j$ at time $t$, such as age, marital status, residing in an urban area (before the pandemic), as well as a set of status/circumstances variables, such as employment status and change in income, that may be associated with the outcome. The fixed-effect ${\gamma }_{jt}$ represents the fixed-effect for the administrative zone $j$ at time $t$. One can consider them as the interaction of time and location fixed effects. These fixed effects control for any factor that may affect the SWB of all individuals in the same administrative zone at the same time (Nov. 2020 or Feb. 2021). These factors include but are not limited to formal and informal institutions, culture, religion, gender norms and attitudes, average demographic characteristics of an administrative zone, economic structures and conditions within an administrative zone, gender gap indicators, institutional qualities (like government capacity), government response to the pandemic, and infrastructure (like the healthcare availability) to name a few. Since administrative zones are within a country, these fixed effects are stronger than country fixed-effects and capture all country-level differences as well. Therefore, we cannot include any country-level variables as covariates, such as gender gap, inequality, cultural norms, and economic, political, and institutional conditions, in our regressions, because the administrative zone-time fixed effects have already controlled for them. In addition, since these administrative zone fixed effects are time-specific, they capture any time-varying variable at the country and sub-country level, such as country and local level policy responses to COVID-19 or the development of the disease over time. Since these fixed effects capture the effect of a myriad of counfounding factors, as described above, they provide a robust, reliable estimate of the effects of individual circumstances, like changes in income or hours worked on SWB. The dataset contains 12 administrative zones in Jordan, 12 in Morocco, 24 in Tunisia, and 27 in Egypt, totaling 75 zones overall. ${\varepsilon }_{ijt}$ is the error term. As we discuss below, Table 5 shows the results of this regression.

## Results and discussion

Some of the most important factors that affect wellbeing are employment status, income, and the burden of work at home (for women, in particular). Therefore, we first review the changes in individuals’ employment status, households’ income, and the burden of work at home during the pandemic (compared to Feb. 2020).

**Fig. 2:**
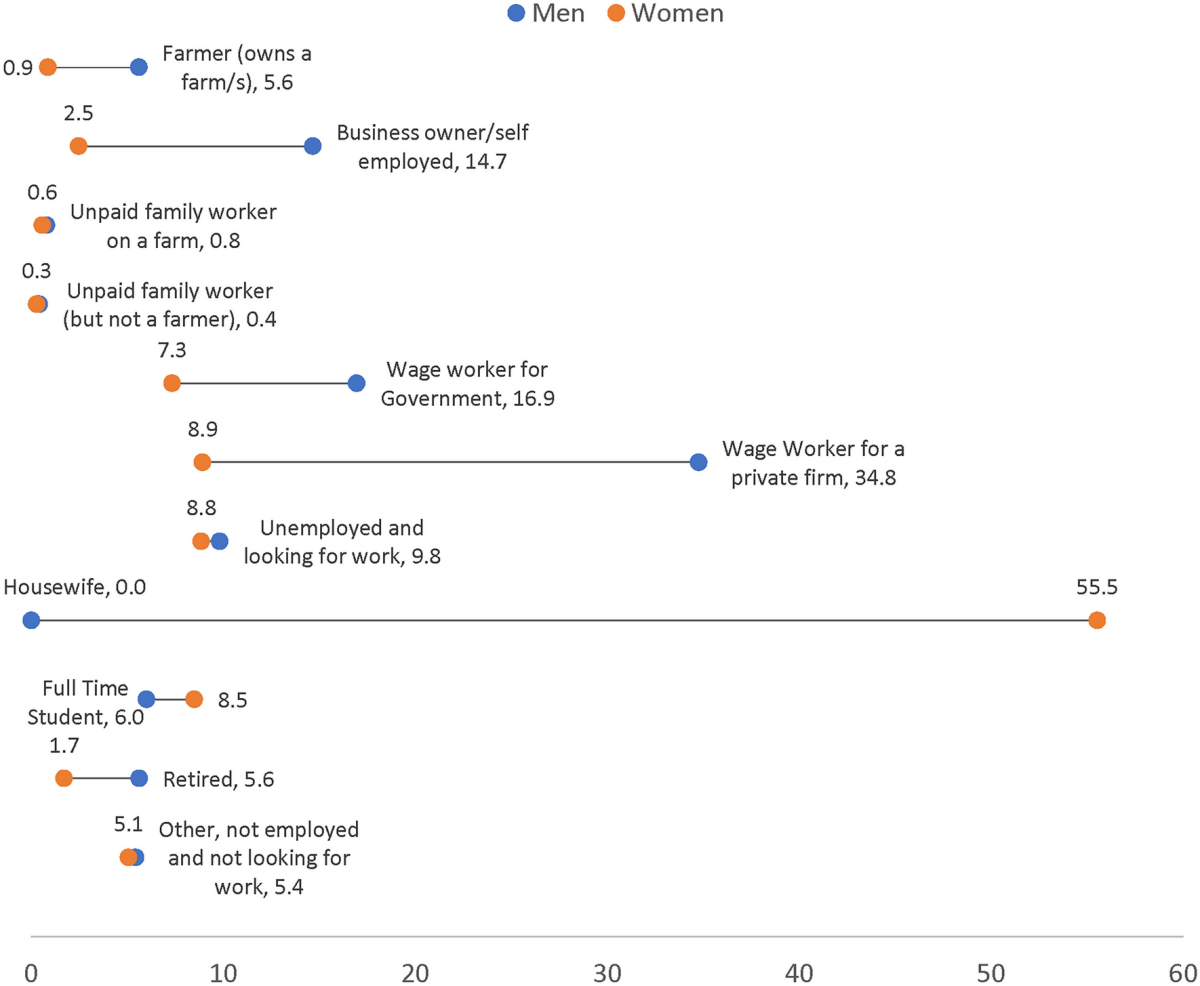
Main job/activity in Feb. 2020 (before the pandemic), by sex. Authors’ calculations based on variable cor18. The sample sizes for women and men are 5778 and 8780, respectively.

Figure 2 lists the shares of respondents who chose various jobs/activities as their main job before the pandemic in all four countries included in the study (The women and men’s sample sizes in this figure are 5778 and 8780, respectively). Over 55% of women were housewives, 8.9% were employed by the private sector, 7.3% were employed by the government, 2.5% were self-employed/business owners, 8.8% were unemployed, 8.5% were full-time students, and about 1% were unpaid family workers. Men, however, were predominantly (34.8%) employed by the private sector, 16.9% of them were employed by the government, 14.7% were self-employed/business owners, 5.6% were farmers (owned a farm), 9.8% were unemployed, 1.2% were unpaid family workers, and 11.5% were out of the labor force.

Panel A of Table 2 shows what share of the sample was employed before the pandemic and in the first and second waves of the survey by sex and country. The largest decline in employment-to-population ratio was experienced by men in Egypt. It was 80% in Feb. 2020 but declined to 59% by June 2020 and eventually rose to 66% in February 2021 (still significantly below its pre-pandemic levels). For women, in comparison, employment-to-population ratios were relatively stable over this period. They declined the most in Morocco by about seven percentage points by November 2020 but went up by five percentage points by Feb. 2021. Interestingly, Tunisian men and women experienced an increase in their employment-to-population ratio during the pandemic (Further analysis shows that 23% of Tunisian women who were not employed before the pandemic but became employed during it chose self-employment/business-ownership, and 70% chose wage-earning in the private sector, the remaining 7% became mostly employed in the government. For Tunisian men, these numbers were 43%, 49%, and 8%. The sample sizes are reported in the table notes).

**Table 2:** Employment-to-population and Unemployment-to-population ratios before and during the pandemic.

	Women			Men		
	Pre-Pand	Wave 1	Wave 2	Pre-Pand	Wave 1	Wave 2
*Panel A—Employment-to-Population Ratios (in %)*						
Egypt	20	19^†^	21	80	59^†^	66
Jordan	15		16	62		57
Morocco	22	15	20	74	69	77
Tunisia	23	27	28	72	74	76
*Panel B—Unemployment-to-population ratios (in %)*						
Egypt	6	4^†^	18	4	6^†^	11
Jordan	7		18	10		18
Morocco	13	16	21	15	19	19
Tunisia	10	19	18	10	10	10

^†^ Wave 1 in Egypt was collected in June 2020, but it was collected in November 2020 in Morocco and Tunisia. So the Egyptian employment to population ratios in wave 1 are not comparable to those of Morocco and Tunisia.

Employment and Unemployment to population ratios in the pre-pandemic time were created based on the variable named cor18 (anyone who did the first six activities in Fig. 2 is considered employed in Feb. 2020 and anyone who reported activity #7 in Fig. 2 is considered unemployed in Feb. 2020.) Employment and Unemployment status in waves 1 and 2 are based on variables named emp and unempsr in the data. Prepandemic variables are emp_Feb20 and unemp_Feb20. The sample sizes for women and men in Jordan are 1218 and 1331, in Egypt are 1400 and 2523, in Morocco are 1511 and 2498, and in Tunisia are 1,649 and 2428, respectively.

The unemployment-to-population ratio, however, significantly increased during the pandemic, particularly for women. Panel B of Table 2 depicts these rates. Egyptian and Jordanian women were particularly hit hard as their unemployment ratios tripled. For Moroccan and Tunisian women, the situation was not substantially better. Their unemployment ratios almost doubled during the pandemic. Among men, Egyptian men were affected the most, while unemployment ratios did not change for Tunisian men.

The rise in unemployment ratios, particularly for women, could be a response to the loss in household income. When household income is reduced, more members of the household enter the labor force to find new sources of income. As a result, unemployment (and potentially employment) ratios may increase. In fact, during the pandemic, 48% of households in Jordan, 41% of households in Egypt, 62% of households in Morocco, and 47% of households in Tunisia experienced a reduction in their income. We do not have the actual income of the households before or during the pandemic in the data but we know whether household income before the pandemic was below or above the median (The approximate income quartile of households are reported in the data, but since these four quartiles are approximate, we prefer to split the households into only two groups: below and above median to get more accurate results). Table 3 reports the percentage of households who experienced a decline in their income by their income category (below and above the median) in Feb. 2020. It shows that a larger share of households whose income was below the median before the pandemic experienced a decline in their income than households whose income was above the median. Some households, particularly in Morocco, did not know or refused to report their income before the pandemic. Therefore, although these results make sense, one should take them with a grain of salt. Because of the large number of missing values for this variable (household income in Feb. 2020), it is not possible to use it in other analyses, for example, regressions.

**Table 3:** Percent of households whose income declined during the pandemic by income group.

Income before the pandemic (Feb. 2020)	Egypt	Jordan	Morocco	Tunisia	All
Below the median	46%	52%	64%	50%	55%
	(1984)	(1541)	(2546)	(1363)	(7434)
Above the median	36%	43%	35%	44%	41%
	(1584)	(886)	(387)	(1764)	(4621)
Don’t know/Refused	31%	47%	61%	49%	55%
	(355)	(122)	(1076)	(244)	(1797)

Total numbers of observations are in parentheses.

**Fig. 3:**
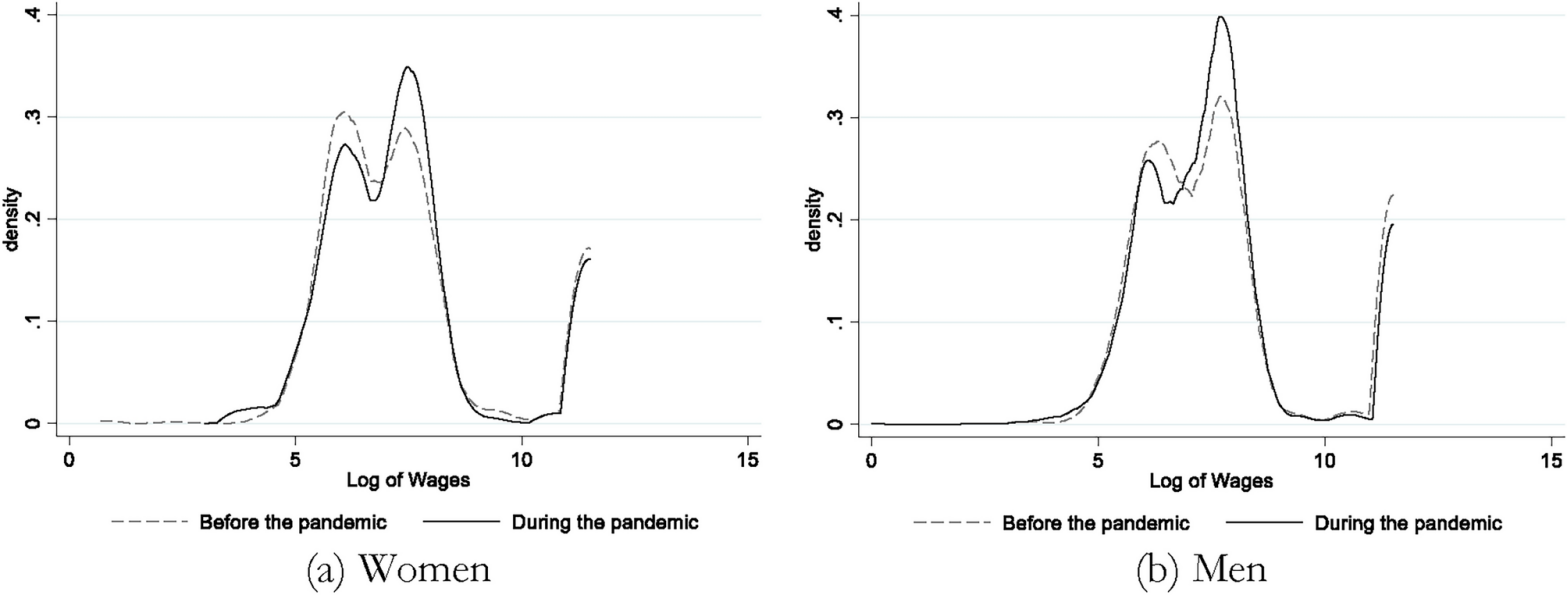
Distribution of log of wages, by sex. Authors’ calculations based on variables named wor7 and wor8. The sample sizes for women and men before the pandemic are 3808 and 1048 and after the pandemic are 4132 and 985, respectively.

Although we do not have households’ actual incomes, the data reports the monthly salary of wage-earners. Figure 3 reports the distribution of wages by sex before and during the pandemic. The wage distributions shifted to the left during the pandemic. Further analysis shows that the average wage for men declined by about 10% and for women by about 15% (these declines are statistically significant at 0.1%.) The decline was slightly larger for men employed in the private sector vs. the government (10% vs. 8%) but it was larger for women employed by the government rather than the private sector (24% vs. 10%).

In addition to the labor market outcomes, we can use the women module in the November 2020 and February 2021 waves of the survey to understand the burden of housework and care for women. Culturally, most men in the countries of our study are not involved in childcare or housework. So, the burden of work falls on women, and the surveys asked such questions from women only (See Appendix Fig. 2 on their care work details). Table 4 shows the percentage of women who spent more time caring for children and doing housework during the pandemic compared to February 2020 (The total sample size in each cell is also reported in parentheses). The results show that about 40% of women increased the time they spent taking care of children during the pandemic. This is quite consistent across countries. In Morocco, however, this number declined to about 28% in February 2021. Similarly, about 30–40% of women reported that they increased the amount of time they spent doing housework during the pandemic. These clearly show that not only did women experience worse labor market outcomes, but they also experienced more burden of work at home.

**Table 4:** Percent of women who spent more hours doing the following activities during the pandemic compared to Feb. 2020 (in %).

Activities	Egypt	Jordan	Morocco		Tunisia	
	Feb. ‘21	Feb. ‘21	Nov. ‘20	Feb. ‘21	Nov. ‘20	Feb. ‘21
Caring for children*	39 (729)	42 (1,218)	44 (836)	28 (675)	41 (824)	37 (825)
Housework	30 (729)	33 (1218)	35 (836)	27 (675)	44 (824)	40 (825)

*Only women who lived in a household with children responded to this question. So we report the percent of women in households with children who spent more time caring for children. Housework, however, is for all women. These questions were not asked in the June 2020 Egyptian survey (Egypt’s first wave.) The total numbers of women are in parentheses.

Considering all the burdens that the pandemic created, particularly for women, it is interesting to see its impact on the SWB of individuals. To understand what factors are associated with the SWB index, we run regressions based on Eq. (1) for the whole sample and several sub-samples. To easily interpret the coefficients of the regressions, we normalize the SWB index by dividing it by its standard error (Which is 1.708659) and use the normalized SWB (NSWB) index as the dependent variable. Hence, the size of the coefficients depicts the change in SWB in terms of standard deviation.

Table 5 reports the results of these regressions. In each regression, we include *Household income decreased*, which is a binary variable equal to one if household income declined relative to Feb. 2020 and zero otherwise, *Unemployed* and *Out of labor force* binary variables, which represent two of the three employment categories (the third one is being employed and is omitted from the regression), *More childcare*, which is a binary variable equal to one if a respondent spent more time caring for children vs. Feb. 2020 and zero otherwise, *More housework*, which is a binary variable equal to one if a respondent did more housework vs. Feb. 2020, education dummies (three binary variables representing *Basic education*, *Secondary education*, and *Higher education*; *Less than basic* is the omitted category), age dummies (three binary variables representing 30–39, 40–49, and 50–64 age groups; 18–29 is the omitted age group), *Married* (a binary variable), *Urban* (a binary variable), and household size. Administrative Zone—Time fixed effects are included to capture any factor that affects all respondents in an administrative zone (within a country) in Nov. 2020 or Feb. 2021. These fixed effects are stronger than country fixed-effects and capture within-country differences as well. They also capture changes over time at the administrative zone level, for example changes in economic conditions, or policy response to COVID-19.

**Table 5:** Regressions of the Normalized SWB Index.

	All	Men	Women	Women (w. hw.)	Women Only		Urban Women only		
					Urban	Rural	Employed	Unemp	Out of LF
	(1)	(2)	(3)	(4)	(5)	(6)	(7)	(8)	(9)
Household income decreased	− 0.210**	− 0.257**	− 0.142**	− 0.136**	− 0.161**	− 0.094	− 0.234**	− 0.101	− 0.207**
	(*p* < 0.001)	(*p* < 0.001)	(*p* < 0.001)	(0.001)	(*p* < 0.001)	(0.240)	(0.023)	(0.205)	(*p* < 0.001)
Unemployed	− 0.252**	− 0.313**	− 0.102	− 0.093	− 0.113	− 0.0374			
	(*p* < 0.001)	(*p* < 0.001)	(0.169)	(0.210)	(0.261)	(0.705)			
Out of labor force	− 0.0517	− 0.0796	0.104	0.107	0.112	0.109			
	(0.261)	(0.283)	(0.190)	(0.178)	(0.216)	(0.393)			
More childcare vs. Feb. 2020				− 0.001	− 0.032	0.138	0.136	− 0.030	− 0.123
				(0.981)	(0.626)	(0.117)	(0.213)	(0.770)	(0.249)
More housework vs. Feb. 2020				− 0.106**	− 0.126**	− 0.049	− 0.053	0.074	− 0.278**
				(0.038)	(0.031)	(0.559)	(0.607)	(0.338)	(0.001)
Basic education	0.089*	0.170**	− 0.025	− 0.023	− 0.075	0.209	− 0.302	− 0.221**	0.0497
	(0.070)	(0.017)	(0.700)	(0.720)	(0.347)	(0.102)	(0.145)	(0.0282)	(0.670)
Secondary education	0.137**	0.207**	0.030	0.038	− 0.017	0.172	− 0.225	0.0920	− 0.00692
	(*p* < 0.001)	(*p* < 0.001)	(0.572)	(0.478)	(0.803)	(0.141)	(0.336)	(0.454)	(0.952)
Higher education	0.158**	0.203**	0.094	0.104	0.083	0.238*	− 0.0176	0.0789	0.0997
	(*p* < 0.001)	(*p* < 0.001)	(0.143)	(0.105)	(0.299)	(0.075)	(0.944)	(0.512)	(0.384)
Other controls	Yes	Yes	Yes	Yes	Yes	Yes	Yes	Yes	Yes
Admin. Zone—Time FE	Yes	Yes	Yes	Yes	Yes	Yes	Yes	Yes	Yes
Number of observations	12,614	7514	5100	5100	3701	1399	955	993	1753

All variables reported in this table are binary variables equal to one if what the variable name represents is true and zero otherwise. “Less than basic education” is the omitted group for education. Other controls include age (three binary variables representing age between 30 and 39, 40 and 49, and 50 and 64; 18–29 is the omitted group), married (a binary variable), urban (a binary variable), and household size. Almost all of these controls are statistically insignificant at 10% level. *P*-values, clustered at the administrative zone level, are in the parentheses. There are 75 administrative zones in the sample.

**p < 0.05, *p < 0.10.

Column (1) contains the result for the whole sample (men and women combined). It shows that a decline in household income is associated with a 0.21 standard deviation decline in the SWB index. That is about a 14.5% decline from the average SWB. Comparing Columns (2) and (3), we observe that the negative association of the lower household income and SWB is larger for men than women (0.26 standard deviation vs. 0.14; that is, 18% vs. 10%). Unemployed men have a lower SWB index compared to employed men, but there was no difference in SWB between unemployed and employed women (Columns 2 & 3). Men and women who are out of the labor force have statistically the same SWB as employed people. Another interesting gendered difference is that more education increases SWB for men only, not women. Women’s SWB does not benefit from more education.

In Column (4), we add two explanatory variables to the sample for women (because these were asked of women only). They are whether a woman spent more time on childcare compared to Feb. 2020 and whether she spent more time on housework vs. Feb. 2020. The coefficient of *household income decreased* in Column (4) remains the same as Column (3), showing that the association between household income and SWB is robust to the inclusion of the burden of work at home. Interestingly, the results show that women who spent more time on housework at home during the pandemoic (compared to Feb. 2020) experienced about 0.1 standard deviation (~ 7%) decline in their SWB. This result is unique to women as almost all men do not engage in housework. This negative association makes sense as household chores are rarely exciting or inspiring. Taking care of children, however, can have positive returns and can be fulfilling. Therefore, although the increase in childcare created a burden for women, particularly mothers, its fulfilling aspect and the motherly love for their children balanced its impact on SWB.

We then explore the results for various subsamples of women. Columns (5) and (6) have urban and rural women, respectively. This is for two reasons: households in urban areas experienced stricter and better-enforced lockdowns. Moreover, households in rural areas could continue working on farms during lockdowns (because farms are not enclosed spaces) but urban households were more likely to lose their source of income as they worked in enclosed offices and factories and hence, more subject to lockdowns and unemployment. The results show that the decline in household income is associated with a decline in SWB for urban women only. We do not find evidence of that in rural areas, potentially because the decline in household income in rural areas may have little to do with the pandemic per se. Additionally, the negative association between the increase in housework and SWB is pronounced only for urban but not rural women (~ 0.13 standard deviation or ~ 9% decline in SWB). In general, the pandemic might not have disrupted rural life at all. Hence, we turn our focus to urban women in Columns (7)–(9) (The sample for rural women does not produce any statistically significant results).

We divide the sample of urban women based on their labor force participation status into three groups: (1) employed, (2) unemployed, and (3) out of the labor force. The results are reported in Columns (7) through (9), respectively. They show that the negative association between the decline in household income and the SWB is only pronounced for urban women who were employed or out of the labor force, but there is no evidence of that for unemployed women (although the coefficient is negative, it is statistically insignificant). This could be because employed women and women who are out of the labor force could not do anything to increase household income to pre-pandemic levels. This lack of agency creates unhappiness. Unemployed women, however, are hopeful that by finding a job, they can compensate for the decline in household income (they have not lost their agency (and hope) yet. Therefore, their SWB was still unaffected. If their hope does not materialize, they have to leave the labor market (and join the women out of the labor force). This may lead to unhappiness about the decline in household income. Analysis of these subsamples also show that the negative association between time spent on housework and SWB exists for women out of the labor force only.

Interestingly, we do not find any association between age, marital status, living in an urban area, and household size and SWB for both men or women. These controls are not reported in Table 5, although included in all regressions.

## Conclusion and policy implications

The COVID-19 pandemic has been a critical juncture that exacerbated gender inequality and exposed the structural violence experienced by women in the contexts in this study. It is a form of violence where women’s SWB is compromised by the daily pressures of the lockdown; income loss; and the demand on their time due to increased care responsibilities. This structural violence has long been normalized in social institutions that bread social injustices at different discursive levels and praxes. The pandemic has further exposed these vulnerabilities. On a peace continuum, the pandemic placed societies farther from a situation of peace to one closer to conflict to borrow from Davenport et al.^7^. This is a serious outcome, one that will need a proportionately long process of reconciliation. The pandemic has threatened the social fabric of society by disrupting access to social services, particularly schools and childcare facilities. This was disproportionately shouldered by women due to gender norms. Intersectionalities of institutionalized inequalities influenced and particularly governed individuals’ experiences and their SWB.

A key contribution of this study is exposing the forms of structural violence that were exacerbated by the pandemic. The pandemic had its toll on the SWB of both men and women. However, women consistently reported lower SWB than men. This gender difference in SWB might have existed prior to the pandemic, however the pandemic has allowed to expose it by further amplifying the stressors associated with it, including the inequitable distribution of the burden of care, economic pressures and the decline in income. The intersectionality of poverty and gender was clearly shown in the data, with poorer women showing the lowest level of SWB. For women, the economic hardships associated with the pandemic have also been compounded by an increase in the time they spent taking care of children during the pandemic. This is quite consistent across the four countries included in the study. The toll of these changes on the SWB of individuals is quite significant. The decline in household income is associated with a 0.21 standard deviation decline in the SWB index. That is about a 14.5% decline from the average SWB. More interestingly, women who had an increase in their housework during the pandemic experienced a 7% decline in their SWB. This was particularly the case for urban women (~ 9%). This is a serious manifestation of gender inequities and the structural violence that women in the region experience, facing the multiple burdens of poor economic prospects; increased care responsibilities and constrained mobility. The lockdown aggravated women’s unequal social status along with their worsened economic opportunities and SWB.

Not surprisingly, the data shows that being unemployed (actively searching for a job) is negatively associated with a lower SWB index (compared to being employed). However, this association is only pronounced for men and does not exist in any of the subsamples of women. This signifies deeply structured gender roles based on male breadwinner norms. Those who are out of the labor force (i.e., not working or actively searching for a job) have no more or less SWB than those who are employed. As opposed to housework, more time spent on taking care of children does not have a negative impact on SWB. As education increases, SWB increases, but only for men. We find hardly any association between education and SWB for women. We also do not find any association between other controls (age, marital status, living in an urban area, and household size) and SWB for both men and women.

The policy approach to mitigate the impact of the pandemic in all four countries, like most countries in the world, did not consider the differential impact of the pandemic on women vs. men and did not address it. However, our analysis on the MENA region confirms a global pattern that shows that women in particular had to bear the brunt of the economic pressures; the increased household demands and the ensuing psychological toll of the pandemic. There is now a sobering recognition that a gendered approach to mitigating the impact of the pandemic was gravely missing^58^. The parameters of this approach would have entailed first and foremost a recognition of the importance of the care economy as central for social reproduction^59^. This brings to the fore the feminist agenda for the need to ‘recognize, reduce and redistribute’ care work (ibid.).

COVID-19 exposed different intersectionalities of inequality across nations, genders, and socio-economic groups. It did so, it subjected more people to structural violence and social injustices. With the conceptualization of positive peace as the absence of structural violence, the pandemic brought more societies closer to conflict on a peace-conflict continuum^7^. It was a time of great intensity with a potentially long-lasting impact on the quality of peace in these societies. The data presented in this paper whistle the need for policies that are particularly focused on women in this part of the world to mitigate the compounded burden of gender inequity and the structural violence associated with it.

## Supplementary Information

Supplementary Information.

## Data Availability

The datasets employed in this study are provided by the Economic Research Forum (ERF) Open Access Micro Data Initative (OAMDI). The citation is COVID-19 MENA Monitor Household Survey (CCMMHH), http://www.erfdataportal.com/index.php/catalog. Version 2.0 of the licensed data files; CCMMHH_Nov-2020-Feb-2021. Egypt: Economic Research Forum (ERF) (2021). They are available in the Economic Research Forum OAMDI data portal at http://www.erfdataportal.com/index.php/catalog#_r = 1,723,217,026,354&collection = &country = &dtype = &from = 1963&page = 1&ps = &sid = &sk = COVID&sort_by = rank&sort_order = desc&to = 2024&topic = &view = s&vk=
