# Supplementary material for: COVID-19’s gendered effect on subjective wellbeing in MENA countries
Source: Sci Rep. 2025 Jan 10;15:1574. doi: 10.1038/s41598-024-84452-7 (PMC11723909; doi:10.1038/s41598-024-84452-7)
Supplement: Supplementary file 1 — Supplementary Information. [file 41598_2024_84452_MOESM1_ESM.docx]

**Online Appendix Figures & Tables**

| Figure A1 – Proportion of women reporting each category of work in the past week (during the pandemic) |
| --- |
|  |
| Note: Authors’ calculations based on variables wom7_1 through wom7_8. The sample size for women with children is 3,324 and for women without children is 1,783. |

| Table A1 – Percent reported various frequencies for how often they felt cheerful and in good spirits during the pandemic, by country and sex (in %) | | | | | | | | | | | |
| --- | --- | --- | --- | --- | --- | --- | --- | --- | --- | --- | --- |
| Activities | Egypt | |  | Jordan | |  | Morocco | |  | Tunisia | |
|  | Women | Men |  | Women | Men |  | Women | Men |  | Women | Men |
| All of the time | 6 | 11 |  | 6 | 10 |  | 14 | 19 |  | 9 | 12 |
| Most of the time | 15 | 10 |  | 18 | 13 |  | 14 | 15 |  | 11 | 8 |
| More than half the time | 4 | 6 |  | 8 | 7 |  | 9 | 8 |  | 7 | 9 |
| Less than half the time | 16 | 15 |  | 10 | 11 |  | 11 | 13 |  | 12 | 14 |
| Some of the time | 36 | 33 |  | 31 | 26 |  | 30 | 27 |  | 35 | 26 |
| At no time | 22 | 24 |  | 28 | 33 |  | 22 | 18 |  | 26 | 31 |
| * Only women who lived in a household with children responded to this question. So we report the percent of women in households with children who spent more time caring for children. Housework, however, is for all women. These questions were not asked in the June 2020 Egyptian survey (Egypt’s first wave.) Sample sizes for women and men in Egypt are 729 and 1,271, in Jordan are 1,218 and 1,331, in Morocco are 1,511 and 2,498, and in Tunisia are 1,649 and 2,428, respectively. | | | | | | | | | | | |

| Table A2 – Percent reported various frequencies for how often they felt calm and relaxed during the pandemic,  by country and sex (in %) | | | | | | | | | | | |
| --- | --- | --- | --- | --- | --- | --- | --- | --- | --- | --- | --- |
| Activities | Egypt | |  | Jordan | |  | Morocco | |  | Tunisia | |
|  | Women | Men |  | Women | Men |  | Women | Men |  | Women | Men |
| All of the time | 5 | 10 |  | 6 | 8 |  | 15 | 19 |  | 8 | 11 |
| Most of the time | 11 | 11 |  | 16 | 15 |  | 14 | 16 |  | 9 | 7 |
| More than half the time | 5 | 9 |  | 8 | 8 |  | 9 | 9 |  | 6 | 9 |
| Less than half the time | 18 | 17 |  | 13 | 14 |  | 9 | 11 |  | 12 | 13 |
| Some of the time | 40 | 33 |  | 29 | 23 |  | 30 | 27 |  | 32 | 25 |
| At no time | 21 | 20 |  | 29 | 32 |  | 22 | 18 |  | 32 | 34 |
| * Only women who lived in a household with children responded to this question. So we report the percent of women in households with children who spent more time caring for children. Housework, however, is for all women. These questions were not asked in the June 2020 Egyptian survey (Egypt’s first wave.) Sample sizes for women and men in Egypt are 729 and 1,271, in Jordan are 1,218 and 1,331, in Morocco are 1,511 and 2,498, and in Tunisia are 1,649 and 2,428, respectively. | | | | | | | | | | | |

| Table A3 – Percent reported various frequencies for how often they felt active and vigorous during the pandemic,  by country and sex (in %) | | | | | | | | | | | |
| --- | --- | --- | --- | --- | --- | --- | --- | --- | --- | --- | --- |
| Activities | Egypt | |  | Jordan | |  | Morocco | |  | Tunisia | |
|  | Women | Men |  | Women | Men |  | Women | Men |  | Women | Men |
| All of the time | 10 | 18 |  | 9 | 11 |  | 15 | 19 |  | 10 | 15 |
| Most of the time | 9 | 11 |  | 20 | 18 |  | 14 | 16 |  | 12 | 9 |
| More than half the time | 9 | 13 |  | 10 | 10 |  | 9 | 9 |  | 8 | 11 |
| Less than half the time | 20 | 17 |  | 13 | 14 |  | 9 | 11 |  | 15 | 14 |
| Some of the time | 34 | 28 |  | 26 | 21 |  | 32 | 27 |  | 30 | 24 |
| At no time | 16 | 14 |  | 22 | 25 |  | 21 | 18 |  | 25 | 27 |
| * Only women who lived in a household with children responded to this question. So we report the percent of women in households with children who spent more time caring for children. Housework, however, is for all women. These questions were not asked in the June 2020 Egyptian survey (Egypt’s first wave.) Sample sizes for women and men in Egypt are 729 and 1,271, in Jordan are 1,218 and 1,331, in Morocco are 1,511 and 2,498, and in Tunisia are 1,649 and 2,428, respectively. | | | | | | | | | | | |

| Table A4 – Percent reported various frequencies for how often they woke up feeling fresh and rested during the pandemic,  by country and sex (in %) | | | | | | | | | | | |
| --- | --- | --- | --- | --- | --- | --- | --- | --- | --- | --- | --- |
| Activities | Egypt | |  | Jordan | |  | Morocco | |  | Tunisia | |
|  | Women | Men |  | Women | Men |  | Women | Men |  | Women | Men |
| All of the time | 7 | 12 |  | 6 | 8 |  | 16 | 19 |  | 13 | 15 |
| Most of the time | 9 | 9 |  | 20 | 19 |  | 15 | 17 |  | 11 | 8 |
| More than half the time | 5 | 11 |  | 8 | 9 |  | 8 | 8 |  | 6 | 9 |
| Less than half the time | 22 | 20 |  | 16 | 13 |  | 9 | 11 |  | 13 | 17 |
| Some of the time | 38 | 30 |  | 26 | 21 |  | 31 | 28 |  | 32 | 23 |
| At no time | 18 | 18 |  | 25 | 30 |  | 22 | 18 |  | 26 | 28 |
| * Only women who lived in a household with children responded to this question. So we report the percent of women in households with children who spent more time caring for children. Housework, however, is for all women. These questions were not asked in the June 2020 Egyptian survey (Egypt’s first wave.) Sample sizes for women and men in Egypt are 729 and 1,271, in Jordan are 1,218 and 1,331, in Morocco are 1,511 and 2,498, and in Tunisia are 1,649 and 2,428, respectively. | | | | | | | | | | | |

| Table A5 – Percent reported various frequencies for how often their daily lives filled with things that interest them during the pandemic, by country and sex (in %) | | | | | | | | | | | |
| --- | --- | --- | --- | --- | --- | --- | --- | --- | --- | --- | --- |
| Activities | Egypt | |  | Jordan | |  | Morocco | |  | Tunisia | |
|  | Women | Men |  | Women | Men |  | Women | Men |  | Women | Men |
| All of the time | 24 | 25 |  | 16 | 15 |  | 12 | 16 |  | 35 | 23 |
| Most of the time | 14 | 14 |  | 19 | 17 |  | 16 | 15 |  | 12 | 11 |
| More than half the time | 11 | 11 |  | 9 | 9 |  | 4 | 6 |  | 12 | 11 |
| Less than half the time | 17 | 16 |  | 12 | 11 |  | 7 | 8 |  | 8 | 16 |
| Some of the time | 26 | 26 |  | 18 | 21 |  | 22 | 19 |  | 21 | 22 |
| At no time | 8 | 7 |  | 25 | 27 |  | 39 | 36 |  | 12 | 17 |
| * Only women who lived in a household with children responded to this question. So we report the percent of women in households with children who spent more time caring for children. Housework, however, is for all women. These questions were not asked in the June 2020 Egyptian survey (Egypt’s first wave.) Sample sizes for women and men in Egypt are 729 and 1,271, in Jordan are 1,218 and 1,331, in Morocco are 1,511 and 2,498, and in Tunisia are 1,649 and 2,428, respectively. | | | | | | | | | | | |

| Table A6 – Summary Statistics for Women and Men in the Sample | | | | | |
| --- | --- | --- | --- | --- | --- |
|  | Women (N = 5,100) | |  | Men (N = 7,514) | |
| Variables | Mean | St. Dev. |  | Mean | St. Dev. |
|  |  |  |  |  |  |
| Subjective wellbeing index | -0.11 | 1.61 |  | 0.08 | 1.77 |
| Normalized subjective wellbeing index | -0.06 | 0.94 |  | 0.04 | 1.04 |
| Household income decreased | 0.52 | 0.50 |  | 0.54 | 0.50 |
| Labor Force Status |  |  |  |  |  |
| Employed | 0.24 | 0.43 |  | 0.69 | 0.46 |
| Unemployed | 0.28 | 0.45 |  | 0.19 | 0.39 |
| Out of labor force | 0.48 | 0.50 |  | 0.12 | 0.32 |
| Education |  |  |  |  |  |
| Less than basic | 0.29 | 0.45 |  | 0.25 | 0.43 |
| Basic | 0.16 | 0.37 |  | 0.19 | 0.40 |
| Secondary | 0.29 | 0.45 |  | 0.34 | 0.47 |
| Higher education | 0.26 | 0.44 |  | 0.22 | 0.41 |
| Age | 37.23 | 12.30 |  | 36.83 | 12.07 |
| Married | 0.68 | 0.47 |  | 0.63 | 0.48 |
| Urban | 0.73 | 0.45 |  | 0.66 | 0.47 |
| Household size | 4.73 | 2.03 |  | 4.97 | 2.54 |
| More childcare vs. Feb. 2020* | 0.27 | 0.44 |  |  |  |
| More housework vs. Feb. 2020* | 0.33 | 0.47 |  |  |  |
|  |  |  |  |  |  |
| Note: Subjective Wellbeing Index is formed from principal component analysis of five variables described in Tables A1-A5. Household Income Decreased is a binary variable that is one if household’ income declined and zero otherwise. Employed, Unemployed, and Out of labor force are binary variables that are equal to one if a respondent was employed, unemployed, or out of labor force, respectively, and zero otherwise. Less than basic, Basic, Secondary, and Higher education are binary variables equal to one if a respondent’s education is at the associated level and zero otherwise. Married is a binary variable equal to one if a respondent was married at the time of the survey and zero otherwise. Urban is a binary variable equal to one if a respondent lives in an urban area.  * More childcare vs. Feb. 2020 and More housework vs. Feb. 2020 are binary variables equal to one if a woman spent more time on these activities in the week prior to the survey relative to Feb. 2020. Since these questions are only asked of women, the number of observations for those is 5,100. | | | | | |
